# Characterizing Antibiotic Regimen Modification Behavior, Patient Characteristics, and Outcomes for Patients with Gram-Negative Bacterial Infections, A Retrospective Single-Center Study

**DOI:** 10.3390/antibiotics13040302

**Published:** 2024-03-27

**Authors:** Jason Yamaki, Mirna Mikhail, Richard Beuttler, Philip Robinson, Emre Yücel, Alexandre H. Watanabe

**Affiliations:** 1 Department of Pharmacy Practice, Chapman University School of Pharmacy (CUSP), Irvine, CA 92618, USA; mmikhail@chapman.edu (M.M.); rbeuttle@chapman.edu (R.B.); 2 Hoag Memorial Hospital, Newport Beach, CA 92663, USA; philip.robinson@hoag.org; 3 Merck & Co., Inc., Rahway, NJ 07065, USAalexandre.watanabe@merck.com (A.H.W.)

**Keywords:** antibiotics, prescribing behaviors, Gram-negative, MDRO, outcomes, stewardship

## Abstract

Few studies describe the frequency of antibiotic regimen modification behaviors in the acute care setting. We sought to ascertain patient and treatment characteristics, details of regimen modification, and clinical outcomes with antibiotic modifications. This retrospective study included patients admitted to Hoag Memorial Hospital from 1 January 2019–31 March 2021 with a complicated infection caused by a Gram-negative organism resistant to extended-spectrum cephalosporins or with the potential for resistance (AmpC producers). A total of 400 patients were included. The predominant sources were bloodstream (33%), urine (26%), and respiratory (24%), including patients with multiple sources. The most isolated organisms were *Pseudomonas* spp. and ESBL-producing organisms, 38% and 34%, respectively. A total of 72% of patients had antibiotic regimen modifications to their inpatient antibiotic regimens. In patients where modifications occurred, the number ranged from one to six modifications. The most common reasons for modifications included a lack of patient response (14%), additional history reviewed (9%), and decompensation (7%). No difference in clinical outcomes was observed based on antibiotic modifications. The numerous changes in therapy observed may reflect the limitations in identifying patients with resistant organisms early on in admission. This highlights the need for more novel antibiotics and the importance of identifying patients at risk for resistant organisms.

## 1. Introduction

Carbapenems are considered one of the last lines of defense in the treatment of infections caused by multidrug-resistant (MDR) Gram-negative bacteria (GNB), including extended-spectrum beta-lactamase (ESBL)- and AmpC beta-lactamase-producing enteric organisms, *Pseudomonas aeruginosa*, *Acinetobacter baumannii*, and other non-lactose fermenting bacteria. The increasing prevalence of these organisms in the patient population coupled with increasing resistance to carbapenems among these organisms has become a public health concern [1]. While progress has recently been made in the development of active antibacterials against the above organisms, there is still a deficit in antimicrobial agents for the varying types and degrees of resistance encountered in Gram-negative organisms.

Due to the severity and/or complexity of GNB infections, patients are often initiated on empiric antibiotic therapy prior to clinicians receiving information on the causative pathogen and its susceptibility to antibiotics. This can lead to a situation wherein the patient is receiving an inappropriate treatment, which has been shown to contribute to worse outcomes [2]. Furthermore, there can be a delay in initiating an appropriate and effective therapy, which increases the risk of poor outcomes in patients with sepsis or septic shock [3,4]. In such cases, when a patient has an ongoing infection and their condition deteriorates or the culture test results indicate the susceptibility of the bacteria to certain antibiotics, clinicians may opt to modify the current treatment or prescribe additional antibiotic agents to specifically target the causative bacteria.

Although studies have been conducted to identify the behavioral and social determinants influencing prescribing practices among physicians and other prescribers [5,6,7,8], there is limited documentation in the current literature regarding the frequency and reasons for changing antimicrobial therapy in acute care hospital settings [9]. However, some data do exist in the outpatient setting [5,6,10,11,12]. Based on clinical practice paradigms, clinicians may change treatment therapies based on patient clinical status changes, for example, if a patient decompensates or does not respond clinically despite antimicrobial treatment. Therapeutic modifications to the patient clinical status can occur in both empiric treatment, when the causative pathogen and its susceptibilities are unknown, and in directed therapy, when the organism and its susceptibilities are known. Therapy changes may also occur once the causative organism’s susceptibility is known, which can lead to escalation, de-escalation, or no change.

The primary objectives of this study were to assess the frequency of antibiotic switch and/or add-on, examine the commonly prescribed empiric therapies in our patient population, and identify the documented or potential reasons for modifying treatment regimens in patients infected with aerobic Gram-negative organisms that are resistant to extended-spectrum cephalosporins (e.g., ceftriaxone) or known to carry chromosomally encoded class C beta-lactamases (AmpC). This cohort of patients was chosen because patients infected with these organisms would likely have an increase in inappropriate empiric treatment, or when preliminary culture results identify a genus known to likely be resistant (e.g., *Pseudomonas* or *Serratia*), physicians may modify therapy. Likewise, many of these organisms have the potential to be MDR organisms for which newer agents such as imipenem/relebactam and ceftolozane/tazobactam are available. Hence, understanding prescribing behaviors and the rationale and reasons behind antibiotic regimen modifications, prescribers may be able to make more informed treatment decisions to facilitate positive outcomes.

The secondary objectives of this study were to determine the timing of antimicrobial treatment switch or add-on during the course of treatment, identify the characteristics associated with regimen switch or add-on, examine the commonly used empiric therapies in our patient population, and compare the outcomes of patients who underwent switch or add-on with those who did not undergo such modifications. Patient outcomes based on antimicrobial switching, microbiologic culture and susceptibility results, and the timing of antibiotic administration would also provide information on treatment approaches that may be useful in the future. Moreover, the data collected from this analysis can offer valuable insights for antimicrobial stewardship programs. They can help identify opportunities for optimizing the treatment of patients with antibiotic-resistant organisms, as well as potential opportunities for the de-escalation and escalation of therapy. 

Specifically, this analysis focused on assessing the frequency of Gram-negative antibiotic treatment modifications, documenting the timing of therapy modifications during the treatment course, exploring the documented or potential reasons behind these regimen modifications, examining the frequency of prescribers documenting their rationale, identifying the most common Gram-negative organisms resistant to extended-spectrum cephalosporins, and evaluating the outcomes of patients affected by these modifications. The main hypothesis was that modifications made during treatment are likely broadening therapy due to the poor response or possible resistance of infecting organisms, primarily occurring during empiric treatment.

## 2. Results

A total of 400 patients were included in the study. Only eight patients had repeat admissions accounting for a total of seventeen admissions, one patient accounted for three admissions, and seven patients only had two admissions. The overall median age of patients was 74.5 and ranged from 19 to 101 years, and 243 (61%) of the patients were male. The median Charlson comorbidity index score was 4 (IQR (interquartile range) 3–6). Most patients presented from home (*n* = 319, 80%), 68 (17%) patients were admitted from a skilled nursing facility, and the remaining patients came from a long-term care facility or were transferred from a different hospital (Table 1). Overall, 194 (48.5%) patients presented with sepsis as defined by the Centers for Medicaid Services (CMS), which can be defined by Systemic Inflammatory Response Syndrome (SIRS). Approximately half of patients had received IV antibiotics or been hospitalized in the past 90 days, and 108 (27%) patients had contracted previous infections with a multidrug-resistant organism. The predominant sources were bloodstream (*n* = 164, 33%), urine (*n* = 128, 26%), and respiratory (*n* = 117, 24%), including patients with multiple sources. Only 18 (4.5%) patients had a concurrent SARS-CoV2 infection. 

The median time from suspected infection to the first antibiotic empiric therapy was 2 h (IQR 1–4), with most initial antibiotic administrations occurring in the Emergency Department. In half of the cases, inpatient empiric regimens were prescribed via order sets. If an order set was not used, 37% of the time, the ED regimen was started inpatient. The median time to an antibiotic with activity against the eventual isolated organism was 3.375 h (IQR 1.5–20) and ranged from 0.25 to 360 h, with 14 (3.5%) patients never receiving an antibiotic with activity against the cultured organism(s) prior to discharge. 

A total of 287 (72%) patients had antibiotic regimen modifications to their inpatient antibiotic regimens. As mentioned above, modifications of ED regimens and changes involving agents specifically for Gram-positive organisms were not included in this definition of “modification”. The total number of antibiotic modifications occurring in these patients was 415, with 80% (*n* = 330) occurring during empiric therapy, and the remaining were modifications in directed therapy. For patients where modifications occurred, the number of changes ranged from one to six modifications. The time to the first modification ranged from 2 to 196 h, with a median time of 23.4 h. Of the initial empiric therapy modifications, 84% (*n* = 122) were considered broadening in the spectrum of activity, and most were based on preliminary culture results mainly due to rapid diagnostic nucleic acid amplification test results (*n* = 71, 58%). Additional reasons for modifications were a lack of patient response (*n* = 17, 14%); additional history reviewed (*n* = 11, 9%); decompensation (*n* = 9, 7%); and a different suspected infection, adverse drug reactions, or undocumented reasons (*n* = 15, 12%). 

Specifically looking at directed therapy, the median time to starting directed therapy was 64 h IQR (43.5–86.5) and the time to first modification in directed therapy was 74 h IQR (46–98). In 59% (*n* = 168) of the 287 patients that had therapy modifications, the modification occurred based on culture and susceptibility results, where, in 51% (*n* = 146) of these cases, the change resulted in a narrower-spectrum agent being used. Changes that occurred while the patient was already receiving directed therapy (a shift from one directed therapy to another) were predominantly de-escalations (*n* = 49, 58%). This primarily happened in patients who remained on the same broad-spectrum empiric therapy despite culture results and subsequently underwent a switch in treatment. For the remaining patients wherein escalation occurred, 27% (*n* = 11) had new culture results become available, leading to modifications; and 15% (*n* = 6) experienced a poor clinical response; and the remaining were modified due to adverse drug reactions (ADRs), convenience of dosing at discharge, or undocumented reasons. Directed treatment was only modified twice at most, which indicates that more regimen changes occur during empiric treatment, which could see up to four different empiric treatments. 

Overall, for empiric and directed therapy modifications, infectious diseases physicians were the most common prescribers to make modifications during treatment (*n* = 201, 70%), and only in 4% (*n* = 16) of medication instances was the rationale not documented by a physician. Patients for which modification occurred were more likely to have a higher number of physician consultants, longer time to active therapy, higher likelihood of being admitted to the ICU, longer duration of antibiotic therapy, shorter times to preliminary culture results, and more likely to have a multidrug-resistant organism (MDRO) isolated. (Table 1).

The most common inpatient initial empiric therapies were piperacillin/tazobactam (*n* = 127, 32%), cefepime (*n* = 94, 24%), ceftriaxone (*n* = 67, 17%), and meropenem (*n* = 59, 15%). The most used antibiotic for directed treatment was ertapenem, followed by meropenem, together accounting for 57% (*n* = 244) of directed treatments used. Directed treatment was continued in 71% (*n* = 195) of patients at discharge, where 29% (*n* = 79) of patients were transitioned to an oral treatment (this change was not counted in regimen changes). The median duration of outpatient-directed treatment was 7 days. Patients that experienced regiment modifications during their inpatient treatment were more likely to have slightly longer median durations of treatment as an outpatient (median (IQR): 7 (5–10) vs. 7 (4–7), *p* = 0.006).

The median time to microbiology culture collection was 2 h (IQR 0.75–19.5) from initial arrival to the hospital. Comparing the time to culture collection between patients that had modifications to those that did not, those without regimen changes had slightly longer median times: 3.75 vs. 1.75 h (*p* = 0.0128). The median time to preliminary results overall was 26.25 h, and the time to preliminary results in patients with regimen modifications was shorter, 23 vs. 40 h (*p* = 0.0004). The time to susceptibility results did not differ between groups and ranged from 22.5 to 554 h with a median of 61.25 h (IQR 47.63–83). The most common organisms isolated were *Pseudomonas* spp. and ESBL-producing organisms, with 38% (*n* = 159) and 34% (*n* = 146) of isolates, respectively. *Escherichia coli* made up the largest portion of ESBL-producing organisms (*n* = 118, 81%), followed by *Klebsiella* spp. (*n* = 19, 13%) and *Proteus* spp. (*n* = 9, 6%). The remaining 28% (*n* = 119) of organisms were comprised primarily of *Enterobacter* spp., *Serratia* spp., and *Citrobacter* spp. Overall, 109 (27%) patients had more than one organism grow out or multiple sites of isolation on cultures. Categorically, 188 (47%) organisms were multidrug resistant (MDR), including to ESBL organisms; 42 (11%) were carbapenem resistant; and 2 organisms were extensively drug resistant. Specifically looking at the 158 *Pseudomonas* spp. isolates, 93 (59%) were pan-susceptible, 22 (14%) were multidrug resistant, and 28 (18%) *Pseudomonas* isolates were carbapenem resistant. Interestingly, of the carbapenem-resistant *Pseudomonas* spp. isolates, 11 (39%) were not considered MDR, with carbapenems being the only beta-lactams they were resistant to. Susceptibility testing for ceftazidime/avibactam was only carried out for 10 organisms overall, and 150 organisms were tested for ceftolozane/tazobactam. Out of the 150 organisms tested for susceptibility to ceftolozane/tazobactam, only 5 were resistant, 4 of which were ESBL *E. coli* and 1 of which was carbapenem-resistant *Klebsiella pneumoniae*. Patients infected with an MDRO were more likely to have their antibiotic regimen modified compared to those that did not, 50% vs. 34% (*p* = 0.0034).

In comparing outcomes between patients that had changes to antimicrobials versus those where no modifications occurred, no statistically significant differences in clinically meaningful outcome measures were observed. These outcomes included 30- and 90-day readmission and 30- and 90-day mortality. Other outcomes included time to clinical stability, which was 1 day, and the median time between the patients where modification occurred vs. not was also 1 day in each group, but with a slightly longer time in IQR (*p* = 0.0128) for the modification group. The longer time to reach clinical stability also led to longer lengths of stay (LOSs) in patients in the modification group with a median LOS of 7 days vs. 5 days in the no modification group (*p* < 0.0001). The 30-day readmission rate for all patients was approximately 21% of patients being readmitted; the majority of the time, this was infection related, with over half of patients being readmitted for the same type of infection, which was usually caused by the same organism (72%). However, as mentioned above, there was no difference between patient groups in terms of 30- or 90-day readmission rates. The 30- and 90-day mortality rates also did not differ between groups (Table 2). 

**Figure 1:**
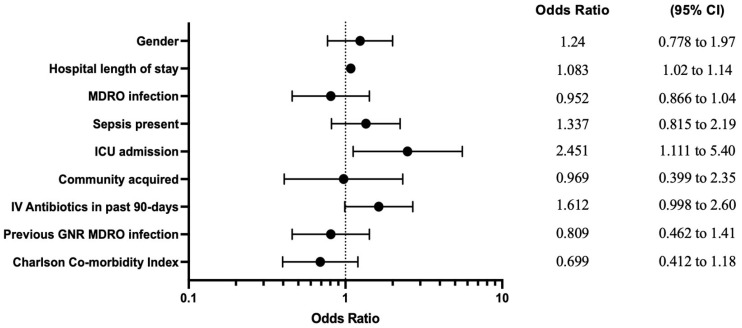
Regimen modification associations. Forest plot of the multiple logistic regression analysis utilizing a generalized estimating equations model, illustrating independent factors associated with antibiotic therapy modification. Calculated odds ratios with 95% confidence intervals are depicted.

Clinical outcomes were assessed for receipt of active antibiotic within 48 h compared to those who received an active antibiotic after 48 h. There was no difference in 30- or 90-day mortality in patients that received an active ABX within 48 h vs. not. A difference was observed in the length of stay for the <48 h group as the median (IQR) was 6 (4–10) vs. 8 (5.75–14.75), *p* = 0.0015 (Table 3). When exploring for variables associated with therapy modifications utilizing multiple logistic regression, ICU admission and hospital length of stay were the variables associated with therapy modification using a mixed-effects model and with a generalized estimating equations (GEE) model. When utilizing the regression model with only index events (removing subsequent visits from patients admitted multiple times, *n* = 382), IV antibiotic use in the previous 90 days became an additional significant variable (Table 4). The GEE model was selected for representation in a forest plot in Figure 1.

## 3. Discussion

This study sought to describe the number of antibiotic regimen modifications patients with Gram-negative infections experienced during their treatment course and reasons for modifications, specifically in patients infected with resistant or potentially resistant organisms. While practicing clinicians are aware that antibiotic regimens undergo frequent changes in the inpatient setting due to various reasons, few data have been published on how often modifications occur, the rationale for modifications, how often the rationale is documented, when in therapy changes often occur, and the associated outcomes with antimicrobial regimen modifications. Previous studies have described antibiotic prescribing characteristics in the outpatient setting; our study adds to the literature by specifically focusing on patients with Gram-negative infections. To our knowledge, this is the first study to examine the frequency, rationale, timing, and associated outcomes with antimicrobial therapy modifications in the inpatient setting in patients with organisms that were resistant or had the potential to be resistant to extended-spectrum cephalosporins. 

In our study, the patient population was predominantly elderly, consistent with the overall demographic composition of our institution. Despite the advanced age of the patients, the overall mortality rate was found to be 9%, a figure lower than reported in previous studies focusing on patients with ESBL-producing organisms [13,14]. The median time to any antibiotic being administered was 2 h, indicating that upon presentation to the Emergency Department, patients were promptly evaluated for infection and received antibiotics. This rapid response aligns with the recommended timeline for initiating antibiotic therapy, particularly for patients with sepsis [15]. The median time to an active antibiotic was considerably longer at ~24 h. This delay can be attributed to the fact that our patient population was infected with organisms that were or had the potential to be resistant to extended-spectrum cephalosporins, including *Pseudomonas* spp. and ESBL organisms. As a result, prescribers or order sets may not have initially included carbapenems or broader-spectrum anti-pseudomonal agents as empiric options. Notably, the time to active antibiotic therapy, though relatively delayed, occurred much earlier than the availability of culture and susceptibility results, which took an average of approximately 61 h from when infection was first suspected. This was primarily due to the availability of rapid diagnostics for blood cultures utilizing nucleic acid amplification testing as part of patient standard of care. When the DNA of resistant pathogens such as *Pseudomonas aeruginosa* or a resistance gene such as CTX-M was detected, the therapy was promptly adjusted to cover for these organisms. This likely explains the relatively short median time to the first antibiotic regimen modification, which occurred at ~23 h, consistent with the time to an active antibiotic and the median time to preliminary microbiology culture results of approximately 26 h. This underscores the crucial role and clinical utility of rapid diagnostics in starting active antimicrobial therapy, particularly for invasive infections, which has previously been described in the literature [16,17,18].

Regimen modification was common with 72% of patients experiencing modification specifically to Gram-negative targeting agents, with most of the modifications occurring during empiric therapy. The changes occurring during empiric treatment are not unexpected as, in practice, microbiology laboratories provide daily, if not multiple times daily, updates to the patient chart with new or preliminary culture results, including rapid diagnostic results, thus leading to empiric therapy adjustments. Furthermore, in line with antimicrobial stewardship best practices, once a patient was on directed therapy, regimen modifications were more likely to narrow treatment regimens. Significantly, the study only accounted for modifications made to antibiotics targeting Gram-negative organisms. It did not encompass regimen changes involving antimicrobials targeting Gram-positive organisms or other microorganisms. Additionally, modifications made to the initial regimen administered in the Emergency Department upon patient admission were also not included in the analysis. Thus, patients would have an even higher number of antimicrobial modifications if these aspects were also included.

Our study examined the implications of antibiotic regimen modifications in the context of MDRO-infected patients, focusing on patient outcomes. We found an association between regimen modifications and MDRO infections, but major patient outcomes such as 30- and 90-day mortality and 30- and 90-day readmission did not show significant differences. However, patients that experienced regimen modifications did have prolonged hospital stays (6 vs. 8 days (*p* < 0.0001), possibly influenced by factors such as inadequate initial therapy, delayed culture results, or the perceived need for extending antibiotic therapy when MDROs were isolated. Further exploration of outcomes based on the initiation of active antibiotics within 48 h of suspected infection revealed that only length of hospital stay and time to clinical stability were significantly different (Table 3). Consistent with what would be expected, patients that received an active antibiotic > 48 h from the onset of a suspected infection had longer lengths of hospital stay, likely due to either not responding to initial empiric therapy that was not active or possibly a delay in preliminary culture results. Bonine et al. used a similar definition of where a delay in therapy was defined as no receipt of an active agent within 2 days of the index date. They found that a delay in active antibiotic therapy was also associated with an increased length of stay and increased hospital cost, as would be expected. Furthermore, in their patient population, an increase in mortality was observed with delay in active antibiotic therapy [19]. Thus, our findings align with the existing literature, reinforcing the notion that delays in initiating active antibiotic therapy can be associated with increased hospital stays and costs, highlighting the critical importance of timely active antibiotic administration in the management of infections.

To determine any factors that might be predictive of patients requiring regimen modifications, multiple logistic regression with various models was used to identify risk factors that could potentially influence therapy modifications, including the MDRO status, which was univariately associated with modifications. In this analysis, the only variables associated with therapy modification were infection-related admission to the ICU and hospital length of stay, per the GEE and mixed-effects models. The representative forest plot (Figure 1) from the GEE model is likely the best fit model for this study population, as we are able to include all patient encounters and the GEE model makes fewer assumptions about the correlation structure between repeated measurements compared to traditional and mixed models [20]. Patient-specific risk factors that would be available to review and act upon at admission to the hospital, including previous antibiotic use and hospitalization in the past 90 days and previous MDRO infection, were included in the multiple logistic regression, but there were no significant associations.

As our study focused on patients with extended-spectrum cephalosporin-resistant or potentially resistant Gram-negative organisms, 47% of the isolates were identified as multidrug-resistant organisms (MDROs). Among these, a significant majority (82%) comprised organisms producing extended-spectrum beta-lactamases (ESBL). MDR *Pseudomonas* spp. constituted a substantial portion of the remaining MDROs; however, notably, 59% of *Pseudomonas* spp. were pan-susceptible. As mentioned above, patients infected with an MDRO were more likely to undergo antibiotic regimen modification, as indicated by univariate comparison. Despite the high prevalence of MDROs, only two patients were infected with carbapenem-resistant Enterobacterales (CRE), and two patients were infected with extensively drug-resistant (XDR) *Acinetobacter* spp. All four of the patients survived at 30 and 90 days, which may not have been expected considering the high mortality rates observed with CRE and XDR organisms, which have been reported to be 30–80%. In the CRE cases, the time to active therapy was within 10 h as the patients were also started on an aminoglycoside because of their previous history of CRE and previous known susceptibility to these agents. In both cases of XDR *Acinetobacter* infection, the time to active treatment was delayed to over 72 h. Overall, nine patients received ceftazidime/avibactam or ceftolozane/tazobactam, which were our formulary antibiotics reserved for the treatment of MDROs and were used in this study for patients with carbapenem-resistant *Pseudomonas* spp. or CRE. While the overall number of patients with CRE or XDR organisms was low during this time period, the observed association with regimen modification, potential increased length of hospital stay, and potential delay in active therapy within this patient population warrant close consideration of the likelihood a patient is infected with an MDRO in order to start prompt active therapy with a newer broad-spectrum antibiotic, such as ceftazidime/avibactam, ceftolozane/tazobactam, or imipenem/relebactam. As multidrug-resistant organisms continue to increase in incidence, including *Clostridioides difficile* infection (CDI), exposure to various antibiotics and antibiotic classes may increase the incidence of both CDI and resistant organism generation. In theory, frequent changing to therapy may lead to exposure to numerous different antibiotics, resulting in potential resistance or increased patient microbiome disruption. However, in practice, changes to antimicrobial therapy frequently occur despite this risk. Importantly, in many instances, modification to antimicrobial regimens is warranted, particularly in patients not responding to therapy or when additional culture information becomes available, which were the primary reasons within our patient population for antimicrobial regimen modifications. However, in our study, the impact on the patient microbiome, CDI risk, and resistance generation were not explored. Future research on the unintended consequences of regimen changes on resistance pressure may be warranted.

As a single-center retrospective observational study, there are inherent limitations associated with these data. This includes the potential generalizability of these data and our findings relevant to other centers both in the US and outside the US. Our patient population was specific to patients infected with extended-spectrum cephalosporin-resistant or potentially resistant organisms and did not include patients solely infected with non-resistant organisms, which can impact generalizability. Limitations associated with free text from medical records may include variability in the quality of documentation by providers in notes and missing information or explicit justification of treatment approaches. Potential inherent selection bias was mitigated by setting a priori enrollment criteria, definitions for exposures and outcomes, and randomization prior to patient selection and review. The outcomes of interest, however, were minimally susceptible to misclassification. Invasive confirmatory culture samples may not always be obtained, and this limits our ability to definitively identify the causative pathogen and susceptibility profile. This limitation mirrors the limitation in clinical practice. However, the inclusion requirement is an infection caused by Gram-negative organisms resistant or with the potential to be resistant to extended-spectrum cephalosporins; thus, this would minimize the potential of missing an organism due to the inability to acquire an invasive culture. Confounding is a potential issue in observational studies; we attempted to minimize this through regression analyses when analyzing factors associated with regimen modifications. Finally, when exploring the secondary objectives of the study in relation to clinical outcomes, these estimates were generated without adjusted models; as such, they are not adjusted estimates. We acknowledge this as a limitation, as adjusting for potential confounding variables would provide a more accurate estimation of the association between regimen modifications and clinical outcomes.

## 4. Materials and Methods

This was a retrospective descriptive study conducted at Hoag Hospital, Orange County, California, USA, a 584-bed community hospital. Hoag hospital has had an antimicrobial stewardship program for the past 16 years. The study included all hospitalized adult patients (aged ≥ 18 years) with complicated Gram-negative infections as defined above (complicated urinary tract infection, bacteremia, intra-abdominal infection, and/or pneumonia), as confirmed by culture, which grew an aerobic Gram-negative extended-spectrum cephalosporin-resistant organism. Organisms included all aerobic Enterobacterales and non-fermenting Gram-negative organisms. Patients were excluded if they did not meet the inclusion criteria or if patients were infected with Gram-positive bacteria only, were co-infected with Gram-positive organisms other than in intra-abdominal infections, did not receive Gram-positive targeting antibiotics within 24 h of intra-abdominal infection onset, received less than 24 h of total treatment, or had less than 48 h of hospitalization. Charts of patients with Gram-negative infections from a microbiology report based on organism and source from 1 January 2019 to 31 March 2021 were reviewed. Patients were randomly chosen by selecting every tenth patient, whose electronic medical record was reviewed for inclusion and exclusion criteria, and all pertinent clinical data were collected if the selected patient met the criteria. Inclusion of patients was not limited to index episodes as patients were still included in the study if they had more than one admission to the hospital during the time period. 

MDR strains were defined as those organisms non-susceptible to an antimicrobial agent in three or more classes of antibiotics: carbapenems, penicillins (piperacillin/tazobactam), cephalosporins (ceftazidime, ceftriaxone, and/or cefepime); monobactams; aminoglycosides; and fluoroquinolones. In cases of Gram-negative polymicrobial infection caused by an extended-spectrum cephalosporin-resistant (ESCR) organism and a non-ESCR isolate, this was defined as having an ESCR organism and the patient included in the study. Infections were defined as nosocomially acquired if the onset of infection was >48 h after admission or community acquired if <48 h after admission. At our hospital, nucleic acid-based rapid diagnostic tests are routinely used in standard of care to provide molecular information within ~3 h of testing a micrology sample. Currently, order sets are available to guide prescribers in the treatment of infections. These order sets were developed based on the type of infection, formulary of antimicrobials, and annual cumulative antibiogram data. Restricted antimicrobials can only be ordered by an Infectious Diseases physician.

In addressing the primary objective, patient data were collected and were analyzed to determine the frequency of switch/add of Gram-negative antimicrobial therapy, the overall time in hours to the start of any empiric antimicrobial therapy, directed antimicrobial therapy, and the timing of when switch/add occurred. When classifying modifications to antimicrobial regimens, the initial ED regimen was not considered an inpatient empiric and, thus, deviations from ED regimens were not classified as modifications. The most used empiric and directed agents were calculated. In patients where switch/add on occurred, the frequency of these changes was calculated based on the reasons for changing therapy: (1) decompensation (worsening of symptoms/vitals leading to a higher level of care), (2) lack of response, (3) culture results, (4) adverse reaction, (5) preliminary culture results, and (6) “other”. 

For the purposes of the secondary objectives, patients were divided into two groups: those that had switch/add occur and those that did not; and patient covariates were compared to identify any associations with therapy switch/add. Patient covariates included age and gender, source of infection, infection type, previous hospital admission and antibiotic use 90 days prior to admission; nosocomial vs. community-acquired infection, comorbid conditions, Charlson Comorbidity Index, ICU admission, and active initial therapy versus delayed active therapy. Outcomes of interest for comparison included duration of antibiotic therapy post culture, length of stay in hospital post culture, discharge disposition, readmission within 90 days, and the composite outcome of in-hospital death or discharge to hospice. 

Continuous variables were compared using the Mann–Whitney U test and the *t*-test. Qualitative categorical variables were compared using the Chi-square test, odds ratios, and 95% confidence intervals (CIs). For univariate analysis of outcomes, adjusted models were not used. Multiple logistic regression analysis was conducted on patient factors potentially associated with switch/add-on and included all statistically significant variables from univariate analysis, gender, age, ICU admission, MDR status, type of infection, and any other clinically relevant variables, whether they were statistically significant or not. Measures of independence were obtained to assess the performance of the models. Backward elimination was used to remove each successive least significant variable. Each variable was then checked by itself using linear regression models. Multicollinearity was assessed amongst covariates. Both a generalized estimating equations model and a mixed-effects model with a random effect of patients to control for multiple admissions by the same patients were utilized [21]. An additional multiple logistic regression was utilized with only index encounters. All three models were compared as part of a sensitivity analysis to look at the effects of the small number of multiple events from the same patients. The analysis was performed with the stepwise logistic regression model of R version 4.3.2, RStudio statistical package.

## 5. Conclusions

A large portion of patients with complicated Gram-negative infections caused by organisms that are resistant or potentially resistant to extended-spectrum cephalosporins had modifications to their antibiotic treatment regimens. Univariate analysis revealed that modifications to antimicrobial regimens were associated with MDR organism isolation, which was also associated with longer lengths of stay. Furthermore, not receiving an active antibiotic within 48 h was also associated with longer lengths of stay. From an antimicrobial stewardship perspective, it is critical to have a balanced approach in ensuring the appropriate patient populations receive optimal therapy, as too narrow a spectrum of agents may be ineffective, leading to detrimental effects on the patient outcomes, while overly broad-spectrum agents may lead to adverse drug reactions [22,23,24,25] and/or the development of resistance. As the prevalence of MDR organisms continues to increase, access to broader and more active agents such as ceftolozane/tazobactam, ceftazidime/avibactam, and imipenem/relebactam and newer agents will become more necessary. Thus, further studies on identifying patient risk factors associated with MDR organisms are necessary to identify patients with MDROs and thus start empiric active agents sooner, as our results demonstrated that this has an impact on hospital length of stay, and in other studies, poor clinical outcomes were associated with delays in active treatment.

## Figures and Tables

**Table 1:** Patient demographics and clinical characteristics.

Variables—Median (IQR ^#^)	Overall *n* = 400 (%) (IQR)	Modifications *n* = 287 (%) (IQR)	No Modifications *n* = 113 (%) (IQR)	*p*-Value
Age (y)	74.5 (64–82)	75 (62–83)	74 (66.5–81.5)	0.99
Male (%)	243 (61)	181 (63)	62 (54.9)	0.1306
Previous hospitalization in past the 90-days	216 (54)	161 (56)	55 (48.7)	0.1798
Previous antibiotics in the past 90-days	192 (48)	145 (50.5)	47 (41.6)	0.1075
Previous GNR MDRO	108 (27)	74 (25.8)	34 (30)	0.3826
COVID-19 infection	18 (4.5)	14 (4.9)	4 (3.5)	0.7894
Residency Prior to Admission				
Home	319 (80)	224 (78)	95 (84)	0.3964
Skilled Nursing Facility	68 (17)	51 (18)	17 (15)	0.5135
Long-Term Acute Care Facility	2 (0.5)	2 (0.01)	0	NS
Other Hospital	10 (2.5)	9 (3)	1 (0.01)	0.2941
Homeless	1 (0.25)	1 (0.003)	0	NS
Sepsis (SIRS, Severe, Shock)	194 (48.5)	147 (51.2)	47 (41.6)	0.0828
ICU admission	74 (18.5)	65 (22.6)	9 (8)	0.0005
Time (h) to active therapy	3.375 (1.5–20)	4 (1.5–24.75)	2.5 (1.5–4.875)	0.002
Time (h) to empiric ABX ED	2 (1–4)	2 (1–3.5)	2.5 (1.5–4)	0.0019
Empiric #1 DOT	2 (2–4)	2 (2–4)	--	--
Time (h) to Empiric #2	23.38 (16.13–42)	23.38 (16.13–42)	--	--
Empiric #2 DOT	2 (2–3)	2 (2–3)	--	--
Total Empiric DOT	3 (3–5)	3 (3–5)	--	--
Time (h) to Directed ABX	64 (43.5–86.5)	64 (43.5–86.5)	--	--
Directed ABX #1 DOT	3 (2–5)	3 (2–5)	--	--
Time (h) from Directed #1 to #2	74 (46–98)	74 (46–98)	--	--
Directed ABX total DOT	3 (2–6)	3 (2–6)	--	--
Total ABX DOT	6 (4–9))	6 (5–10)	5 (4–7)	<0.0001
ABX changes	1 (0–2)	1 (0–2)	--	--
MD consults	2 (1–3)	2 (2–3)	2 (1–3)	0.0098
Charlson Co-morbidity Index	4 (3–6)	4 (3–6)	4 (3–6)	0.6402
ABX at Discharge DOT	7 (5–10)	7 (5–10)	7 (4–7)	0.0060
Time (h) to culture send out	2 (0.75–19.5)	1.75 (0.75–10.5)	3.75 (0.25–26)	0.0128
Time (h) to prelim result	26.25 (17–51)	23 (16.5–47)	40 (22–66)	0.0004
Time (h) to culture and susceptibility	61.25 (47.63–83)	60 (46.3–80.7)	63.88 (49.25–92)	0.1381
Number of ABX classes culture was resistant to	5 (2–9)	2 (0–6)	1 (0–3)	0.0082
MDRO organism isolated	181 (45.3)	143 (49.8)	38 (33.6)	0.0034

^#^ IQR—interquartile range, comparison between modification and no modification groups; ABX—antibiotics; DOT—days of therapy; MDRO—multidrug-resistant organism; ED—Emergency Department; ICU—intensive care unit; NS = not significant; *p*-value ≤ 0.05 = statistically significant.

**Table 2:** Patient outcomes based on regimen modification.

Patient Outcomes	Overall *n* = 400 (%)	Modifications *n* = 287 (%)	No Modifications *n* = 113 (%)	*p*-Value
Days to clinical stability (IQR)	1 (0–2)	1 (0–3)	1 (0–2)	0.0137
Hospital LOS (IQR)	6 (4.25–10)	7 (5–11)	5 (4–8)	<0.0001
ICU admission	74 (18.5)	65 (22.6)	9 (8)	0.0005
30-day mortality	36 (9)	28 (9.8)	8 (7.1)	0.4450
90-day mortality	7 (1.8)	4 (1.4)	3 (2.7)	0.4086
30-day readmission	85 (21.3)	55 (19.1)	30 (26.5)	0.1041
90-day readmission	130 (32.5)	91 (31.7)	39 (34.5)	0.5896

LOS—length of stay; *p*-value ≤ 0.05 = statistically significant.

**Table 3:** Outcomes by receipt of active antibiotic within 48 h of suspected infection.

Patient Outcomes	<48 h *n* = 342 (%)	≥48 *n* = 44 (%)	*p*-Value
Days to clinical stability (IQR)	1 (0–2)	0 (0–1)	0.0056
Hospital LOS (IQR)	6 (4–10)	8 (5.75–14.75)	0.0015
30-day mortality	29 (8.48)	4 (9.09)	0.7704
90-day mortality	6 (1.75)	1 (2.27)	0.5555
30-day readmission	76 (22.22)	5 (11.36)	0.1160
90-day readmission	41 (11.99)	3 (6.81)	0.4498

*p*-value ≤ 0.05 = statistically significant.

**Table 4:** Multiple logistic regression analysis.

	Regression (Mixed-Effects Model)		Regression (GEE Model)		Regression (Index Only Model)	
Variable	Odds Ratio	95% CI	Odds Ratio	95% CI	Odds Ratio	95% CI
Charlson Co-morbidity Index	0.690	0.397 to 1.2	0.699	0.412 to 1.18	0.648	0.377 to 1.11
Previous GNR MDRO infection	0.807	0.457 to 1.42	0.809	0.462 to 1.41	0.829	0.467 to 1.43
IV antibiotics in past 90-days	1.63	0.991 to 2.69	1.612	0.998 to 2.60	1.64	1.02 to 2.68
Community acquired	0.973	0.407 to 2.32	0.969	0.399 to 2.35	0.949	0.410 to 2.31
ICU admission	2.49	1.12 to 5.56	2.451	1.111 to 5.40	2.83	1.30 to 6.86
Sepsis present	1.35	0.814 to 2.23	1.337	0.815 to 2.19	1.26	0.772 to 2.07
MDRO infection	0.951	0.861 to 1.05	0.952	0.866 to 1.04	0.955	0.867 to 1.05
Hospital length of stay	1.08	1.02 to 1.14	1.083	1.02 to 1.14	1.08	1.02 to 1.14
Gender	1.24	0.768 to2.00	1.24	0.778 to 1.97	1.21	0.753 to 1.94

## Data Availability

Data are unavailable due to privacy and/or ethical restrictions.
